# Mental Health Workers’ Knowledge and Attitude Towards Borderline Personality Disorder: A Saudi Multicenter Study

**DOI:** 10.7759/cureus.31938

**Published:** 2022-11-27

**Authors:** Enas M Aljohani, Buthainah D Aldawood, Samaher A Alnajdi, Ayman A Alamri, Raafat Shuqdar

**Affiliations:** 1 Faculty of Medicine, Taibah University, Medina, SAU; 2 Clinical Psychology, King Saud Bin Abdulaziz University, Riyadh, SAU; 3 Psychiatry, Taibah University, Medina, SAU

**Keywords:** borderline personality disorder, saudi arabia, stigma, mental health workers, bpd

## Abstract

Background

Borderline personality disorder (BPD) is a mental illness characterized by emotional instability. Its prevalence can be as high as 1.8% among the general population. Poor knowledge and negative perceptions of the disorder by mental health workers (MHWs) can affect patients' care and their help-seeking behavior. This study aims to explore MHW's knowledge and attitudes toward BPD.

Method

A cross-sectional study was conducted on MHWs across the five regions of Saudi Arabia (SA) using a questionnaire that assessed knowledge, attitude, and training regarding BPD.

Results

Data collected from 1028 MHWs showed a good knowledge level. Superior knowledge was observed among females, residents in the central region, physicians, those who received specific BPD training, and MHWs who had more experience and frequent interactions with BPD patients. Participants had moderate to high levels of perceived knowledge and confidence regarding the identification, assessment, and management of BPD patients. Undergraduate training programs were the most reported source of information on the disorder. While 66% of participants admitted that they find dealing with BPD patients more difficult and thought patient management was inadequate, 71% were willing to attend further BPD training.

Conclusion

MHWs in SA have moderate knowledge of but negative perceptions of BPD; specific training is needed to improve the care provided for BPD patients.

## Introduction

Among the general population, borderline personality disorder (BPD) prevalence is believed to be 0.2-1.8%. However, it can be as high as 20% among psychiatric inpatients and 10% among psychiatric outpatients [1]. According to the Diagnostic and Statistical Manual of Mental Disorders, Fifth Edition (DSM-5), BPD is "a pervasive pattern of emotional dysregulation, impulsiveness, an unstable sense of identity, and difficult interpersonal relationships" [2].

In comparison to other personality disorders (PDs), BPD has received considerable attention in the psychiatric and psychological literature [3]. BPD can be associated with several psychiatric and medical comorbidities [4,5]. A study conducted in 2017 affirmed that a diagnosis of BPD in adolescence is associated with severe impairments in health-related quality of life and psychopathological distress [6].

BPD is one of the most stigmatized mental disorders [7]. Mental illness stigma refers to the prejudice and discrimination directed toward this group of patients [8]. By understanding the causes and impact of stigma, it may be possible to reduce its negative effect on patients with mental illness, thus improving their help-seeking behavior and enhancing their treatment engagement [9,10].

Several studies have discussed the causes and effects of this stigma [10,11]. Among other psychiatric conditions, including other PDs, BPD was associated with a negative perception, even by mental health workers (MHWs) [11]. This stigma seems to be more prevalent among nursing staff compared to other MHWs [12]. Terms such as "difficult," "treatment resistant," "manipulative," "demanding," etc., were found to be used by MHWs to describe BPD patients [13]. These terms most likely resulted from certain characteristics of BPD that may slow therapy progress. For example, intense anger, chronic suicidal ideation, self-injury, recurrent suicide attempts, and fluctuating levels of functioning [14]. Furthermore, patients with BPD are noted to be especially sensitive to rejection and may react to perceived abandonment with self-harm or by withdrawing from treatment [13,14].

Help-seeking is a well-established healthy coping strategy that can assist BPD patients to cope with severe distress [15]. Studies have demonstrated that current treatment and health care workers' (HCWs) responses are often inadequate and fail to meet patients’ needs, especially in emergency situations [7,16].

When MHWs' attitudes towards BPD patients are assessed, more than 80% view them as difficult to work with, and indeed, more difficult to treat than patients with other mental disorders [17]. In another study, MHWs stated having strong negative emotions (e.g., feelings of frustration, helplessness, and anger) towards BPD patients [18,19].

A study conducted in Australia showed that staff was generally knowledgeable of the disorder, although few of them had received specific training concerning BPD. However, 95% were willing to attend further BPD education or training [17]. More recent studies have shown that a brief training program for mental health staff can improve their understanding and perception of the disorder [20,21]. Another study, from 2018, showed that there has been a significant improvement in clinicians’ attitudes toward BPD during the last 15 years [22].

In Saudi Arabia (SA), limited information is available regarding PDs when compared to depression or anxiety, which are both extensively addressed in Saudi literature. The Saudi National Mental Health Survey conducted in 2020 did not include PDs or BPD [23,24]. Due to the limited available data, the current study offers valuable insight into the current state of PDs and BPD in the Saudi healthcare system, as it aims to assess the level of knowledge and training regarding BPD among MHWs in SA and their perception of the disorder and its associated factors.

## Materials and methods

Study design

A cross-sectional study was conducted between July and October 2022 to assess the current level of knowledge and attitudes of MHWs toward BPD in the five regions of SA. The study protocol and instrument were revised by the institutional review board at King Salman bin Abdulaziz Medical City, Medina. The research was unconditionally approved on August 15, 2022 (study ID: 22-053).

Sample population

The study used a convenience sampling method for recruitment. The sample included all MHWs who filled out the questionnaire and matched the inclusion criteria, which includes MHWs (psychiatric residents, registrars, consultants, general practitioners (GPs), family physicians, nurses working in psychiatric settings, social workers, psychologists, and occupational therapists) who were currently working in SA and had direct contact with psychiatric patients. Exclusion criteria included MHWs working outside of SA or those who were not in direct contact with psychiatric patients. A pilot study was conducted on 20 MHWs to check the readability and understanding of the questionnaire, and changes in the questionnaire's formatting were made accordingly. Pilot study responses were not included in the final study sample.

Measurements and tools

A questionnaire was written in both Arabic and English and designed using Google Forms. The questionnaire consisted of four parts, namely, an optional part, a compulsory part, and two close-ended questions. The first part contained informed consent; the second part consisted of a set of questions regarding participants’ sociodemographic information. The third part contained an assessment of participants' knowledge and understanding of BPD based on the DSM-5 criteria [2]. The fourth part contained a scale to assess participants’ attitudes toward BPD using a scale adapted from Cleary et al. [17].

Data collection

To ensure good sample distribution and coverage of all Saudi regions, the sample was divided into five groups according to the Saudi regions (the eastern province, the southern province, the central province, the northern province, and the western province).

Data collectors from the five regions were allocated to collect data from MHWs in each region. Each data collector who completed the assigned number of responses (25) received a letter signed by the principal investigator describing his or her role in data collection, a copy of the research protocol, and a copy of the IRB's ethical approval.

The responses were reviewed daily by the authors to ensure quality. In addition, the data collectors were asked to complete the responses through personal interviews with MHWs or by sending them through professional WhatsApp groups. Posting the questionnaire on social media networks (e.g., Twitter, Facebook, etc.) was prohibited to ensure the quality and reliability of the responses.

Ethical considerations

All subjects gave informed consent to participate in the study, and confidentiality and privacy were respected. A thorough explanation of the study's goals and objectives was provided to ensure their cooperation.

Scoring

In the current study, the knowledge score was calculated by summing up the correct responses to 13 items (each participant scored 1 for each correct answer). Therefore, the score of each participant ranged between 0 and 13, with higher scores corresponding to greater levels of knowledge.

Statistical analysis

Statistical analysis was conducted using RStudio (R version 4.1.1, RStudio, Boston, MA). Descriptive statistics were calculated for categorical variables (frequency and percentages) and continuous variables (median and interquartile range [IQR]). Factors associated with participants’ knowledge were assessed using a Wilcoxon rank sum test or a Kruskal-Wallis rank sum test. The significantly associated variables from the association analysis were subsequently used as independent variables in a multivariate linear regression analysis to explore the independent predictors of higher knowledge scores. The results of the regression analysis were presented as beta coefficients (β) and 95% confidence intervals (95% CIs). Statistical significance was considered at p < 0.05.

## Results

Demographic and occupational characteristics

Initially, 1146 responses were received. However, 118 records did not meet the eligibility criteria (i.e., students, MHWs working outside SA, and those who were not in direct contact with psychiatric patients). Therefore, 1028 records were ultimately analyzed. More than half of the participants (52.8%) were males, while 48.1% were aged ≤30 years. Residents of the Central and Western regions represented 32.0% and 26.8% of the sample, respectively. Regarding occupational characteristics, the most common title was physician (32.5%), of whom the proportion of GPs was 32.7% and that of psychiatric residents was 28.4%. Additionally, psychologists and nurses constituted 28.8% and 21.0% of the participants, respectively. Most respondents were working in clinical duties (84.1%), and 39.7% of them were working in psychiatric hospitals. More than one-third of the participants had <2 years of experience in mental health (Table 1).

**Table 1:** Demographic and occupational characteristics of the participants *The variable has 19 missing values. ^¥^Calculations are based on the responses of 334 physicians (7 missing values).

Parameter	Category	N (%)
Gender	Male	543 (52.8%)
	Female	485 (47.2%)
Age group*	30 years or less	485 (48.1%)
	31–40 years	419 (41.5%)
	41–50 years	105 (10.4%)
Region of residence	Central region	329 (32.0%)
	Eastern region	113 (11.0%)
	Western region	276 (26.8%)
	Northern region	95 (9.2%)
	Southern region	215 (20.9%)
Years of practice in mental health	<2 years	398 (38.7%)
	2–5 years	301 (29.3%)
	6–10 years	198 (19.3%)
	11–15 years	83 (8.1%)
	15 years	48 (4.7%)
Place of work	Community Health Center	218 (21.2%)
	Psychiatric hospital	408 (39.7%)
	Psychiatric unit in a general hospital	295 (28.7%)
	Private mental health center	50 (4.9%)
	Emergency department	1 (0.1%)
	Others	56 (5.4%)
Work duties	Clinical	865 (84.1%)
	Administrative	156 (15.2%)
	Both	7 (0.7%)
Job title	Physician	334 (32.5%)
	Psychologist	296 (28.8%)
	Social worker	96 (9.3%)
	Occupational therapist	66 (6.4%)
	Nurse	216 (21.0%)
	Others	20 (1.9%)
Physician category^¥^	Family medicine resident	27 (8.3%)
	Family medicine registrar	20 (6.1%)
	Family medicine consultant	9 (2.8%)
	Psychiatric resident	93 (28.4%)
	Psychiatric registrar	40 (12.2%)
	Psychiatric consultant	31 (9.5%)
	General practitioner	107 (32.7%)

Experience with patients and training in BPD

Half of the participants (50.5%) were in regular contact with BPD patients (from 1-2 times per week to 1-2 times per month). However, only 14.8% of the respondents had received specific training for the care of patients with BPD. Of those, 61.8% had received clinical training, and 20.4% had attended dedicated courses. Furthermore, the majority (67.8%) had received the training in the last two years. Notably, 59.0% of the participants admitted that BPD patients are inadequately managed. The perceived reasons for inadequate management included a shortage of management services (69.5%), patients being difficult to treat (48.9%), and a lack of training and/or experience (40.2%, Table 2).

**Table 2:** Previous experience and training in borderline personality disorder BPD: borderline personality disorder. *The variable has 132 missing values. ^¥^Calculations are based on the responses of 152 participants who had received specific training for the care of patient BPD. ^§^Calculations are based on the responses of 607 participants who indicated that patients with BPD were inadequately managed.

Parameter	Category	N (%)
Frequency of being in contact with a patient with BPD*	Never	158 (17.6%)
	Once a year or less	125 (14.0%)
	5–6 times a year	160 (17.9%)
	1–2 times a month	270 (30.1%)
	1–2 times per week	183 (20.4%)
Received specific training for the care of patients with BPD	Yes	152 (14.8%)
Type of training^¥^	Clinical training	94 (61.8%)
	Course	31 (20.4%)
	Lectures	15 (9.9%)
	Workshop	12 (7.9%)
Time since receiving the training^¥^	In the last 2 years	103 (67.8%)
	2–5 years ago	37 (24.3%)
	More than 5 years ago	12 (7.9%)
How adequately do you consider that your patients who have a diagnosis of BPD are managed?	Adequately	421 (41.0%)
	Inadequately	607 (59.0%)
Perceived reasons for inadequate management^§^	The patients themselves are very difficult to treat	297 (48.9%)
	There is a shortage of services to treat this patient group	422 (69.5%)
	You lack training and/or expertise	244 (40.2%)
	Others	14 (2.3%)

Knowledge score and the factors associated with knowledge

The detailed responses of the participants are displayed in Table 3. The median (IQR) knowledge score was 9.0 (7.0 to 11.0), with a range between 0 and 13. Based on the rank sum test, the overall knowledge score was associated with all the demographic and occupational characteristics, including participants’ gender (p = 0.003), age (p = 0.006), region of residence (p < 0.001), years of practice in mental health (p < 0.001), place of work (p = 0.022), work duties (p < 0.001), and job title (p < 0.001). Additionally, the knowledge score differed significantly based on the completion of specific training for the care of BPD patients (p < 0.001), the frequency of being in contact with BPD patients (p < 0.001), and self-perceptions of the adequate management of BPD patients (p < 0.001, Table 4).

**Table 3:** Participants’ responses to the knowledge-specific items BPD: borderline personality disorder. *The variable has 5 missing values. ^¥^The variable has 1 missing value. ^§^Indicates correct responses.

Parameter	Category	N (%)
Frantic efforts to avoid real or imagined abandonment*	Disagree	125 (12.2%)
	Agree^§^	687 (67.2%)
	Do not know	211 (20.6%)
A pattern of unstable and intense interpersonal relationships characterized by alternating between extremes of idealization and devaluation^¥^	Disagree	125 (12.2%)
	Agree^§^	790 (76.9%)
	Do not know	112 (10.9%)
Markedly and persistently unstable self-image or sense of self	Disagree	146 (14.2%)
	Agree^§^	716 (69.6%)
	Do not know	166 (16.1%)
Impulsivity in at least two areas that are potentially self-damaging	Disagree	129 (12.5%)
	Agree^§^	762 (74.1%)
	Do not know	137 (13.3%)
Recurrent suicidal behavior, gestures, threats, or self-mutilating behavior	Disagree	141 (13.7%)
	Agree^§^	733 (71.3%)
	Do not know	154 (15.0%)
Affective instability due to a marked reactivity of mood	Disagree	112 (10.9%)
	Agree^§^	802 (78.0%)
	Do not know	114 (11.1%)
Chronic feelings of emptiness	Disagree	184 (17.9%)
	Agree^§^	648 (63.0%)
	Do not know	196 (19.1%)
Inappropriate, intense anger or difficulty controlling anger	Disagree	110 (10.7%)
	Agree^§^	799 (77.7%)
	Do not know	119 (11.6%)
Transient, stress-related paranoid ideation or severe dissociative symptoms	Disagree	153 (14.9%)
	Agree^§^	700 (68.1%)
	Do not know	175 (17.0%)
Patients with BPD should not be hospitalized	Disagree^§^	427 (41.5%)
	Agree	391 (38.0%)
	Do not know	210 (20.4%)
Short-term psychotherapy can be useful to manage crises in patients with BPD	Disagree	428 (41.6%)
	Agree^§^	412 (40.1%)
	Do not know	188 (18.3%)
Antidepressant medication is of no benefit to depression experienced by people with BPD	Disagree^§^	429 (41.7%)
	Agree	367 (35.7%)
	Do not know	232 (22.6%)
A significant number attain some stability in their 30s and 40s	Yes	484 (47.1%)
People with a BPD have a high incidence of depression	Yes	402 (39.1%)
BPD can progress to schizophrenia	Yes^§^	458 (44.6%)
May have short-lived psychotic episodes	Yes	306 (29.8%)

**Table 4:** Factors associated with participants’ knowledge of borderline personality disorder BPD: borderline personality disorder, IQR: interquartile range.

Parameter	Category	Median (IQR)	p-value
Gender	Male	8.0 (6.0, 10.0)	0.003
	Female	9.0 (7.0, 11.0)	
Age group	30 years or less	9.0 (6.0, 10.0)	0.006
	31–40 years	9.0 (7.0, 11.0)	
	41–50 years	8.5 (6.0, 10.0)	
Region of residence	Central region	8.0 (6.0, 10.0)	<0.001
	Eastern region	9.0 (7.0, 10.0)	
	Western region	9.0 (7.0, 10.0)	
	Northern region	7.0 (5.0, 9.0)	
	Southern region	10.0 (8.0, 11.0)	
Years of practice in mental health	<2 years	8.0 (6.0, 10.0)	<0.001
	2–5 years	9.0 (7.0, 11.0)	
	6–10 years	9.0 (7.0, 11.0)	
	11–15 years	10.0 (8.0, 11.0)	
	15 years	8.0 (4.8, 9.2)	
Place of work	Community health center	8.0 (6.0, 11.0)	0.022
	Psychiatric hospital	9.0 (7.0, 11.0)	
	Psychiatric unit in a general hospital	9.0 (7.0, 11.0)	
	Private mental health center	8.0 (5.0, 10.0)	
	Emergency department	6.0 (6.0, 6.0)	
	Others	8.0 (5.0, 9.0)	
Work duties	Clinical	9.0 (7.0, 11.0)	<0.001
	Administrative	7.0 (4.0, 9.2)	
	Both	8.0 (6.5, 10.0)	
Job title	Physician	9.0 (7.0, 11.0)	<0.001
	Psychologist	9.0 (7.0, 11.0)	
	Social worker	8.0 (5.0, 10.0)	
	Occupational therapist	8.0 (5.0, 9.8)	
	Nurse	9.0 (6.0, 10.0)	
	Others	7.0 (1.0, 8.0)	
Received specific training for the care of patient BPD	No	9.0 (6.0, 10.0)	<0.001
	Yes	10.0 (8.0, 11.0)	
Frequency of being in contact with a patient with BPD	Never	7.5 (4.0, 9.8)	<0.001
	Once a year or less	9.0 (6.0, 11.0)	
	5–6 times a year	9.0 (7.0, 10.2)	
	1–​​​​​​​2 times a month	9.0 (7.0, 11.0)	
	1–​​​​​​​2 times per week	9.0 (6.8, 11.0)	
Adequate management of BPD patients	Inadequately	9.0 (7.0, 11.0)	<0.001
	Adequately	8.0 (6.0, 10.0)	

The analysis of independent predictors of higher knowledge scores showed that knowledge scores were higher among women (β = 0.53, 95% CI [0.15, 0.91], p = 0.006), as well as those residing in the central region (β = 0.92, 95% CI [0.20, 1.65], p = 0.013), eastern region (β = 1.64, 95% CI [0.79, 2.49], p < 0.001), western region (β = 1.27, 95% CI [0.54, 2.00], p < 0.001), and southern region (β = 1.97, 95% CI [1.23, 2.71], p < 0.001). Compared to participants with the lowest level of experience working in mental health (<2 years), higher knowledge scores were reported among those with an experience of 2-5 years (β = 0.62, 95% CI [0.13, 1.11], p = 0.013), 6-10 years (β = 0.96, 95% CI [0.32, 1.60], p = 0.003), and 11-15 years (β = 1.35, 95% CI [0.50, 2.20], p = 0.002). Furthermore, respondents who were working in clinical settings had independently higher knowledge scores (β = 1.25, 95% CI [0.68, 1.82], p < 0.001). Compared to physicians, knowledge scores were significantly lower among social workers (β = −0.87, 95% CI [−1.65, −0.91], p = 0.027), occupational therapists (β = −1.03, 95% CI [−1.81, −0.25], p = 0.009), nurses (β = −0.88, 95% CI [−1.42, −0.33], p = 0.002), and other job categories (β = −3.06, 95% CI [−4.41, −1.72, p < 0.001). Knowledge scores were independently higher among participants who had received specific training for the care of BPD patients (β = 0.84, 95% CI [0.27, 1.41], p = 0.004) and those who had self-perceptions of inadequate management of BPD patients (β = 0.45, 95% CI [0.05, 0.85], p = 0.027). Compared to those who were not in contact with BPD patients, higher knowledge was also independently associated with a higher frequency of being in contact with BPD patients, including those who were in contact with them once a year (β = 1.34, 95% CI [0.66, 2.01], p < 0.001), 5-6 times per year (β = 1.64, 95% CI [0.79, 2.49], p < 0.001), 1-2 times per month (β = 1.64, 95% CI [0.79, 2.49], p < 0.001), and 1-2 times per week (β = 1.64, 95% CI [0.79, 2.49], p < 0.001, Table 5).

**Table 5:** Results of the regression analysis of the predictors of higher knowledge scores BPD: borderline personality disorder, CI: confidence interval.

Parameter	Category	Beta	95% CI1	p-value
Gender	Male	—	—	
	Female	0.53	0.15, 0.91	0.006
Age group	30 years or less	—	—	
	31–40 years	−0.26	−0.74, 0.22	0.282
	41–50 years	−0.35	−1.15, 0.45	0.389
Region of residence	Northern region	—	—	
	Central region	0.92	0.20, 1.65	0.013
	Eastern region	1.64	0.79, 2.49	<0.001
	Western region	1.27	0.54, 2.00	<0.001
	Southern region	1.97	1.23, 2.71	<0.001
Years of practice in mental health	<2 years	—	—	
	2–5 years	0.62	0.13, 1.11	0.013
	6–10 years	0.96	0.32, 1.60	0.003
	11–15 years	1.35	0.50, 2.20	0.002
	15 years	−0.13	−1.33, 1.06	0.825
Place of work	Community health center	—	—	
	Psychiatric hospital	0.14	−0.39, 0.67	0.599
	Psychiatric unit in a general hospital	0.01	−0.55, 0.56	0.975
	Private mental health center	−0.93	−1.89, 0.02	0.056
	Emergency department	−3.23	−8.70, 2.23	0.246
	Others	−0.84	−1.70, 0.01	0.052
Work duties	Administrative	—	—	
	Clinical	1.25	0.68, 1.82	<0.001
	Both	0.79	−1.54, 3.12	0.505
Job title	Physician	—	—	
	Psychologist	−0.11	−0.60, 0.38	0.664
	Social worker	−0.87	−1.65, −0.10	0.027
	Occupational therapist	−1.03	−1.81, −0.25	0.009
	Nurse	−0.88	−1.42, −0.33	0.002
	Others	−3.06	−4.41, −1.72	<0.001
Received specific training for the care of patients with BPD	No	—	—	
	Yes	0.84	0.27, 1.41	0.004
Frequency of being in contact with a patient with BPD	Never	—	—	
	Once a year or less	1.34	0.66, 2.01	<0.001
	5–6 times a year	0.96	0.31, 1.61	0.004
	1–2 times a month	1.17	0.58, 1.76	<0.001
	1–2 times per week	1.05	0.41, 1.69	0.001
Adequate management of BPD patients	Adequately	—	—	
	Inadequately	0.45	0.05, 0.85	0.027

Perceived knowledge and confidence in participants’ practice and their contributing roles for BPD patients

In general, the perceived knowledge of the participants was rated as moderate to high for the identification (37.5%), assessment (37.8%), and management of BPD (47.3%). Similarly, participants’ perceived confidence in the identification, assessment, and ongoing management was moderate to high among 33.0%, 35.4%, and 46.6% of the participants, respectively. Less than half of the respondents were moderately to highly confident in their awareness of (43.7%) and referral to specialist services for BPD (36.8%). Approximately two-thirds of the respondents declared that it is moderately to extremely difficult to deal with BPD patients (67.8%) and difficult to deal with BPD patients compared to other patients (61.5%, Table 6). Notably, approximately two-thirds of the respondents stated that they consider themselves to have roles in the assessment (69.5%), management (59.9%), referral (67.7%), and education of BPD patients (69.7%, Table 7).

**Table 6:** Perceived knowledge and confidence BPD: borderline personality disorder.

Parameter	Category	N (%)
Knowledge about the identification of BPD	Very low	272 (26.5%)
	Low	370 (36.0%)
	Moderate	277 (26.9%)
	High	109 (10.6%)
Knowledge about the assessment of BPD	Very low	289 (28.1%)
	Low	351 (34.1%)
	Moderate	260 (25.3%)
	High	128 (12.5%)
Knowledge about the management of BPD	Very low	206 (20.0%)
	Low	336 (32.7%)
	Moderate	308 (30.0%)
	High	178 (17.3%)
Confidence in the identification of BPD	Very low	315 (30.6%)
	Low	373 (36.3%)
	Moderate	239 (23.2%)
	High	101 (9.8%)
Confidence in the assessment of BPD	Very low	278 (27.0%)
	Low	386 (37.5%)
	Moderate	251 (24.4%)
	High	113 (11.0%)
Confidence in ongoing management of BPD	Very low	203 (19.7%)
	Low	346 (33.7%)
	Moderate	297 (28.9%)
	High	182 (17.7%)
Confidence in awareness of specialist services for BPD	Very low	254 (24.7%)
	Low	325 (31.6%)
	Moderate	289 (28.1%)
	High	160 (15.6%)
Confidence in referral to specialist services for BPD	Very low	330 (32.1%)
	Low	320 (31.1%)
	Moderate	254 (24.7%)
	High	124 (12.1%)
How difficult do you find dealing with patients who have a BPD?	Easy	22 (2.1%)
	Neither difficult nor easy	127 (12.4%)
	Slightly difficult	182 (17.7%)
	Moderately difficult	534 (51.9%)
	Very difficult	163 (15.9%)
How difficult do you find dealing with patients who have a BPD compared to other patients?	Less difficult	109 (10.6%)
	The same	287 (27.9%)
	More difficult	632 (61.5%)

**Table 7:** The perceived roles of participants for borderline personality disorder patients BPD: borderline personality disorder.

Parameter	Category	N (%)
The assessment of patients with BPD	No	177 (17.2%)
	Yes	714 (69.5%)
	Unsure	137 (13.3%)
The management of patients with BPD	No	228 (22.2%)
	Yes	616 (59.9%)
	Unsure	184 (17.9%)
The referral of patients with BPD	No	193 (18.8%)
	Yes	696 (67.7%)
	Unsure	139 (13.5%)
Educating and providing information to patients with BPD	No	171 (16.6%)
	Yes	717 (69.7%)
	Unsure	140 (13.6%)

Staff resources

The most helpful resources upon which the respondents rely while working with BPD patients were the education they had received during undergraduate education or training (66.2%), specialist services for BPD patients (61.8%) and the standard protocols for BPD management (55.9%). Of note, the majority of participants (71.5%) were willing to receive further education or training if provided.

## Discussion

BPD is known to be one of the most stigmatized mental illnesses. While this stigma is mainly attributed to the challenging nature of the disorder, it is more distinct among MHWs [22]. This study included a sample of 1028 participants. One study done on MHWs in SA by AlHadi et al. included a similar number of participants (n=1253) [25]. However, others included much smaller samples ranging between 18 and 392 [26-29].

Only half of the participants were in regular contact with BPD patients; this might be attributed to the fact that 21.2% of them work in community health centers. While BPD prevalence is noted to be higher in primary health care centers than in the general population, practitioners without specific mental health training may not be able to correctly identify the symptoms [30,31]. Furthermore, 15% of the participants had only administrative duties, so regular contact with BPD patients was much less likely for these individuals.

In comparison to the findings reported by Cleary et al. [17], where 32% (n = 229) of the participants had received specific BPD training, only 14% of the participants in this study had received such training. This can be explained by the substantial difference in sample size between the two studies. Also, this study sample includes GPs and family physicians, while specific PD and BPD training is usually preserved for HCWs who specialize in mental health [32].

Knowledge of BPD was found to be better among female participants. However, when two studies assessed MHWs' knowledge of electrical convulsive therapy and telepsychiatry in SA, no association between gender and knowledge score was reported [27,29]. This might be explained by the fact that BPD is more prevalent among females, and therefore, females recognize BPD symptomatology more often [33].

Surprisingly, younger MHWs (<40 years old) had higher BPD knowledge scores. This unexpected result may be due to the more up-to-date knowledge of the younger generations along with progressive improvements in medical education and training [29].

The leading mental health facilities in SA are in the central region. Also, training programs in the central region are well established and widely available in contrast to other regions (the northern region specifically was associated with lower knowledge scores). Therefore, working in the central region was independently associated with higher knowledge scores [34].

The knowledge score was found to be lower among non-physicians, including nurses. This finding is consistent with previous research indicating that nurses have particularly negative perceptions of BPD. Developing a better understanding of the disorder can improve this perception and, consequently, the care provided [35]. Suggestions for specific training programs directed at nursing staff have been proposed [35,36].

Two-thirds of the participants believed BPD patients were inadequately managed. While this percentage is similar to the result reported by Cleary et al. [17], it was expected to be higher considering the time gap between the two studies and the major differences in the mental health system between SA and Australia. Notably, those participants who believed the current BPD management to be inadequate had superior knowledge of the disorder. In this study, MHWs had moderately strong perceived knowledge and confidence regarding the identification, assessment, and management of BPD and the difficulty of working with BPD patients. Additionally, they reported that working with BPD patients is more difficult than working with other patients. These results were not surprising, as the negative perception of BPD has been consistently reported in the literature [18,37]. However, Cleary et al. reported high levels of perceived knowledge, confidence, and the ability to deal with patients. Again, this can be explained by the difference in the sampling method between the two studies as well as differences in mental health systems.

The most obvious finding to emerge from the current analysis is that having completed training specifically concerning BPD was associated with higher knowledge scores. This observation is supported by extensive research that has suggested that BPD-specific education and training are necessary to enhance clinicians’ knowledge and management of BPD patients [21,38].

Undergraduate education was the most reported type of training by MHWs, and two-thirds thought it would be the most helpful when dealing with BPD patients. However, researchers showed that brief training courses and workshops were associated with improvements in the clinician's knowledge and perception of BPD [20,38,39]. Moreover, a course regarding the neurobiological basis of BPD also demonstrated attitude and knowledge change [40]. These findings must encourage the start of brief BPD training for MHWs in SA, especially as the majority of participants were interested in attending further training.

As a cross-sectional study, the limitations of this study include the use of an online questionnaire and data collectors. Despite the quality measures implemented, the possibility of recall bias and misinterpretation is still present. Also, the validity of the used questionnaire is unknown because, as far as we know, there is no valid scale to assess knowledge or attitude toward BPD. Further research to assess its validity is suggested.

## Conclusions

MHWs in SA were moderately knowledgeable of BPD. Females, physicians, MHWs working in the central region, and those who had specific BPD training, showed higher knowledge of BPD. However, the majority of MHWs believed that BPD patients were poorly managed and challenging to treat. Notably, 70% expressed a desire to participate in specialist BPD training if it were to be offered. As negative perception and poor knowledge can affect patients' management and outcomes, additional educational and training initiatives are advised. Finally, more research is urged given the scant information currently available regarding the state of PDs in SA.
